# Recurrent Pleuropericardial Effusions Following Amplatzer Amulet Left Atrial Appendage Occlusion: A Case of Post-Cardiac Injury Syndrome

**DOI:** 10.7759/cureus.117289

**Published:** 2026-10-01

**Authors:** Josephine Reece, Brooke Shannon, Sarah Sofka

**Affiliations:** 1 Department of Medicine, West Virginia University School of Medicine, Morgantown, USA

**Keywords:** amplatzer amulet, cardiac tamponade, left atrial appendage occlusion, pericardial effusion, pericarditis, pleural effusion, post-cardiac injury syndrome

## Abstract

Post-cardiac injury syndrome (PCIS) is an inflammatory pleuropericardial syndrome that may follow myocardial, pericardial, or iatrogenic cardiac injury. Although pericardial effusion is a recognized complication of left atrial appendage occlusion (LAAO), recurrent pleuropericardial inflammation after LAAO is less commonly described. We report a patient with persistent atrial fibrillation who underwent uncomplicated LAAO with a 28-mm Amplatzer Amulet device (Abbott Medical, Plymouth, MN, USA) following a recent hemorrhagic stroke. Post-implantation transesophageal echocardiography demonstrated appropriate device positioning without peridevice leak or pericardial effusion. Within two weeks, the patient developed chest pain, dyspnea, electrocardiographic ST-segment elevations, markedly elevated CRP, and a large hemorrhagic pericardial effusion with tamponade requiring drainage of 780 mL of frank blood. The subsequent course was characterized by recurrent unilateral left exudative pleural effusions requiring repeated thoracenteses and chest tube drainage, followed by recurrence of both pleural and pericardial effusions. Serial microbiologic and cytologic studies were negative, and imaging demonstrated no evidence of malignancy. Colchicine was discontinued following an interval hospitalization, after which pleuropericardial effusions and systemic inflammation recurred. Following pleural drainage and reinitiation of colchicine, CRP declined from 87.8 mg/L (<8 mg/L) to 22.5 mg/L (<8 mg/L), respiratory symptoms resolved, and the patient was discharged home on room air. The temporal relationship to LAAO, recurrent pleuropericardial involvement, inflammatory response, exclusion of alternative etiologies, and clinical course were consistent with PCIS. This case highlights PCIS as an important consideration in patients presenting with recurrent pleural or pericardial effusions after LAAO while emphasizing the need to exclude mechanical device complications.

## Introduction

Percutaneous left atrial appendage occlusion (LAAO) provides an alternative strategy for stroke prevention in selected patients with nonvalvular atrial fibrillation for whom long-term anticoagulation is contraindicated or carries substantial bleeding risk. Several transcatheter LAAO systems have been developed, with the Watchman and Amplatzer Amulet representing the two most frequently used devices globally [1]. Although contemporary LAAO is generally safe, pericardial effusion and tamponade remain important procedural complications. In a prospective cohort of 1,023 attempted LAAO procedures, 1,014 devices were successfully implanted, and pericardial effusion occurred in 4.3%, with most occurring early after the procedure; late effusions occurring eight to 365 days after implantation were substantially less common [2].

Post-cardiac injury syndrome (PCIS) encompasses inflammatory pericardial syndromes occurring after myocardial infarction, cardiac surgery, trauma, or invasive cardiac procedures [3,4]. PCIS is thought to result from an inflammatory response to injury of cardiac and pericardial tissues. In the setting of an intracardiac procedure such as LAAO, transseptal instrumentation, manipulation of the thin-walled left atrial appendage, or device-related microtrauma may produce microscopic tissue injury or bleeding into the pericardial space. Tissue injury and the presence of blood within the pericardium can initiate innate and adaptive inflammatory pathways, resulting in pericardial inflammation with secondary involvement of the adjacent pleura. Thus, an initially mechanical injury may serve as the trigger for a subsequent inflammatory pleuropericardial syndrome [3-5].

Recurrent inflammatory pleural effusion has previously been reported following LAAO with the Watchman device, with microperforation and subsequent inflammatory pleuropericarditis proposed as the underlying mechanism [6]. Delayed hemorrhagic pericardial effusion, erosion, and perforation have also been reported following Amplatzer Amulet implantation [7-9]. We describe a case of hemorrhagic pericardial tamponade followed by recurrent unilateral exudative pleural effusions and recurrent pericardial effusion shortly after Amulet implantation, with a clinical course consistent with PCIS.

## Case presentation

An 85-year-old male patient with persistent atrial fibrillation underwent elective LAAO following a recent left basal ganglia hemorrhagic stroke. The medical history was also significant for prior cerebrovascular disease, heart failure with preserved ejection fraction, nonobstructive coronary artery disease, tachy-brady syndrome with dual-chamber pacemaker placement in July 2022, hypertension, hyperlipidemia, and obstructive sleep apnea.

A 28-mm Amplatzer Amulet device (Abbott Medical, Plymouth, MN, USA) was implanted via right femoral venous access. Post-implantation transesophageal echocardiography (TEE) demonstrated a well-positioned device meeting the Compression, Lobe Position, Orientation, Separation, Elliptical Disc (CLOSE) criteria with no visible peridevice leak (Table 1). The interatrial septal puncture was inferior and midseptal with mild continuous left-to-right shunting. Importantly, no pericardial effusion was visualized immediately following the procedure.

**Table 1:** Amplatzer Amulet CLOSE criteria. LAA, left atrial appendage; LCX, left circumflex artery

Criterion	Anatomical and imaging target	Clinical objective
Compression (C)	Proper sizing check of the device lobe within the LAA landing zone	Ensures adequate device tension and stability to prevent embolization
Lobe Position (L)	Lobe should be situated at or distal to the LCX	Confirms the main anchoring mechanism is properly deep inside the appendage
Orientation (O)	The lobe axis should align coaxially with the neck centerline of the LAA	Prevents device tilting, uncovering gaps, or causing uneven structural stress
Separation (S)	Clear separation visible between the internal lobe and the sealing disc	Indicates the device is not overly compressed or deformed longitudinally
Elliptical Disc (E)	The proximal disc must be concave/elliptical and fully seal the ostium	Ensures total closure of the LAA mouth to completely eliminate residual flow

Hospitalization no. 1 with hemorrhagic cardiac tamponade

The patient was subsequently hospitalized nine days after the procedure with acute hypoxic respiratory failure, dyspnea, chest pain, and concern for ST-elevation myocardial infarction. Electrocardiography demonstrated ST-segment elevations in leads V3-V6, I, and II; however, serial troponin measurements were negative. The presenting ECG is shown in Figure 1. Transthoracic echocardiography (TTE) demonstrated a large circumferential pericardial effusion with hemodynamic findings compatible with cardiac tamponade, including a dilated inferior vena cava with less than 50% inspiratory collapse (normal >50% inspiratory collapse) and an estimated right atrial pressure of 15 mmHg (0-5 mmHg).

**Figure 1:**
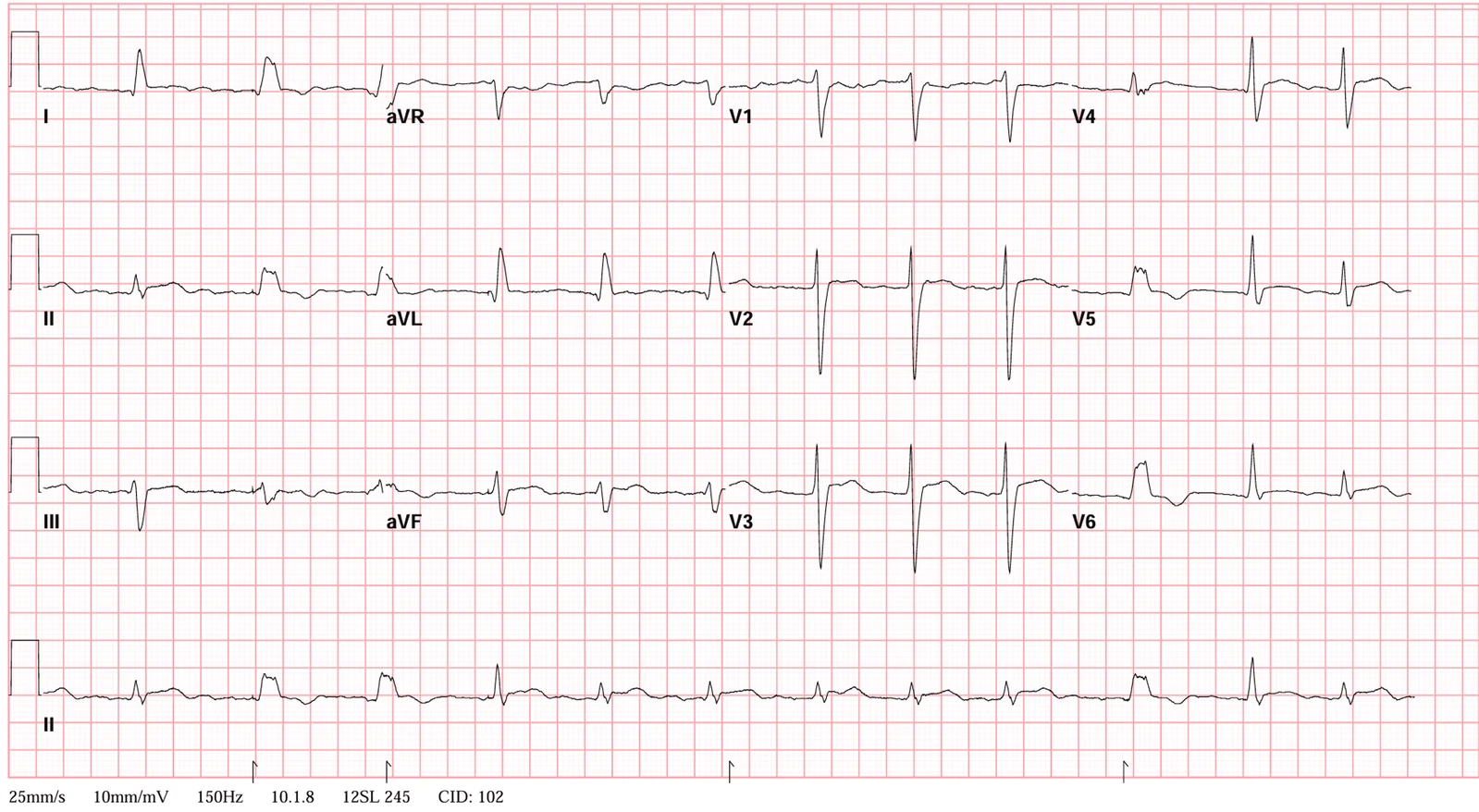
ECG obtained during the initial presentation with pericarditis. Twelve-lead ECG obtained during the first hospitalization demonstrating ST-segment elevations in multiple leads, including leads I, II, and V3-V6. Serial troponin measurements were negative, and the clinical presentation was ultimately attributed to acute pericarditis in the setting of a large pericardial effusion.

Urgent pericardiocentesis yielded 780 mL of frank blood. Pericardial fluid contained 700,000 red blood cells/µL and 1,389 nucleated cells/µL, with a lactate dehydrogenase (LDH) concentration of 2,344 U/L and a protein concentration of 5.5 g/dL. The differential cell count consisted of 33% neutrophils, 34% lymphocytes, 32% monocytes, and 1% eosinophils. Of note, the institution’s lab does not provide reference ranges for body fluid cell counts or chemistry. His CRP was 78.8 mg/L (<8 mg/L) on admission and increased to 103.5 mg/L over the next 48 hours.

The combination of chest pain, multilead ST-segment elevations with negative troponins, hemorrhagic pericardial effusion, and systemic inflammation raised concern for pericardial inflammation following the recent LAAO procedure. High-dose aspirin was initiated for pericarditis, and colchicine 0.6 mg twice a day was subsequently added after infection was considered unlikely.

The patient simultaneously developed a large left pleural effusion. CT scan of the chest on day 3 of admission demonstrated a large layering left pleural effusion with complete collapse of the left lower lobe and lingula, as well as a small circumferential pericardial effusion with the pericardial drain in place. A TTE performed the same day demonstrated resolution of the pericardial effusion but persistence of the left pleural effusion.

The pleural effusion required drainage twice during the admission on hospital days 6 and 9. The fluid was exudative by Light’s criteria. With the first thoracentesis, the pleural fluid contained 2,010 nucleated cells/µL and was predominantly neutrophilic (63%). On the second thoracentesis, three days later, the fluid contained 1,100 nucleated cells/µL and demonstrated a more lymphocyte-predominant profile, with 47% lymphocytes and 3% neutrophils. Pleural fluid cultures were negative, and cytology demonstrated reactive mesothelial cells, histiocytes, and inflammatory cells without evidence of malignancy. Serial chest radiographs demonstrate the large left pleural effusion before drainage and marked interval improvement following drainage in Figure 2A, 2B.

**Figure 2:**
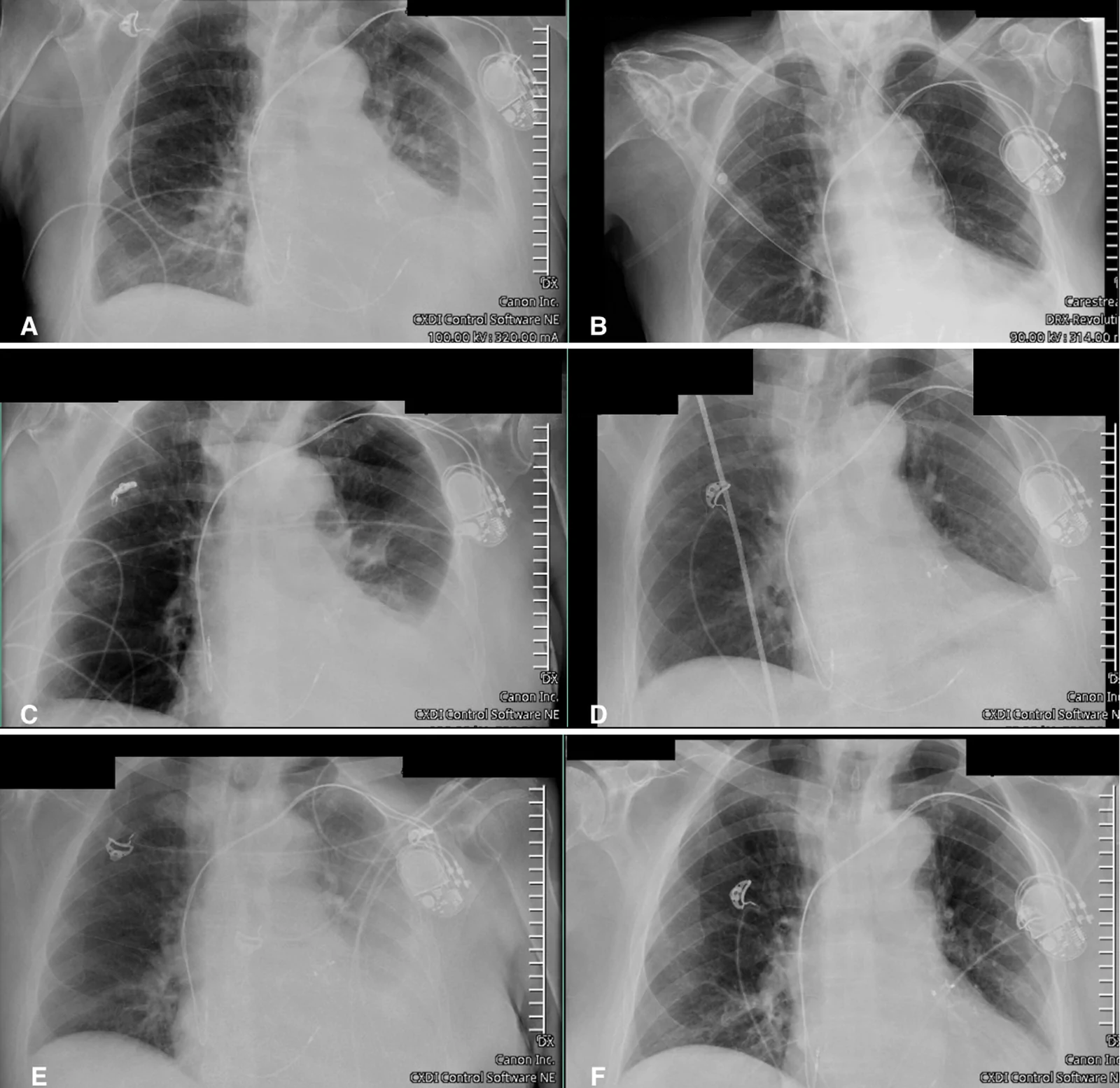
Serial chest radiographs demonstrating recurrent left pleural effusion and response to drainage. (A, B) Chest radiographs from the first hospitalization demonstrating a large left pleural effusion before drainage (A) with marked interval improvement following drainage (B). (C, D) Chest radiographs from the second hospitalization demonstrating recurrent large left pleural effusion before thoracentesis (C) with interval improvement following drainage (D). (E, F) Chest radiographs from the third hospitalization demonstrating recurrent large left pleural effusion before pigtail catheter drainage (E) with marked interval improvement following drainage (F).

Inflammatory markers declined during treatment, with CRP decreasing from 103.5 mg/L (<8 mg/L) on day 3 to 91.9 mg/L on day 4, 21.5 mg/L on day 8, and 13.9 mg/L on day 9. Repeat TTEs on days 3 and 11 demonstrated no residual pericardial effusion. The patient was ultimately discharged to a skilled nursing facility on colchicine 0.6 mg twice a day with a planned duration of three months for pericarditis and cardiology follow-up in two weeks.

Hospitalization no. 2 with recurrent left pleural effusion

The patient was rehospitalized from late July, 10 weeks after the initial LAAO procedure, through early August after a fall and was again found to have a large left pleural effusion. Colchicine was not reported as a home medication. He did not make it to his cardiology follow-up appointment after the last discharge, as he was still in the skilled nursing facility. He remained in the facility for three and a half weeks and had been home for three weeks prior to this hospitalization. It was unable to be determined if colchicine was stopped during the skilled nursing facility admission or at home. A TTE on day 5 of this hospitalization demonstrated preserved left ventricular systolic function with an ejection fraction of 61.6%, normal right ventricular size and function, and a normal inferior vena cava with greater than 50% inspiratory collapse (normal >50% inspiratory collapse), corresponding to an estimated right atrial pressure of 3 mmHg (0-5 mmHg). A trivial pericardial effusion and persistent left pleural effusion were present.

Thoracentesis on day 8 of admission removed 1,500 mL of serosanguineous fluid. Chest radiographs again demonstrated recurrence of the left pleural effusion with interval improvement following thoracentesis (Figure 2C, 2D). The fluid remained exudative and contained 93,748 red blood cells/µL and 378 nucleated cells/µL, with 45% lymphocytes, 21% monocytes, 17% mesothelial cells, 11% eosinophils, and 6% neutrophils. CRP was again markedly elevated at 105.9 mg/L (<8 mg/L). Microbiologic studies remained negative, and cytology again showed reactive and inflammatory cells without malignancy. Colchicine was not included among the patient’s medications at discharge.

Hospitalization no. 3 with recurrent pleural and pericardial effusions

Ten days after discharge, now three months after the LAAO procedure, the patient presented again with shortness of breath and tachycardia and was admitted to our hospital. A CT scan of the chest demonstrated a large left pleural effusion with near-complete atelectasis of the left lower lobe as well as an increased moderate pericardial effusion. CRP was 87.8 mg/L (<8 mg/L) on admission.

Colchicine was restarted on day 2 of admission following cardiology consultation at a dose of 0.6 mg twice a day, with a planned treatment duration of three months for recurrent pericardial inflammation. A TTE that day demonstrated a small circumferential pericardial effusion without echocardiographic evidence of tamponade and a persistent left pleural effusion. Because of the recurrent large pleural effusion, a pigtail catheter was placed. Pleural fluid remained exudative but had evolved to a markedly lymphocyte-predominant inflammatory profile, with 79% lymphocytes, 11% neutrophils, 8% mesothelial cells, and 2% monocytes. CRP was 56.1 mg/L (<8 mg/L), and the erythrocyte sedimentation rate was 43 mm/hr (1-20 mm/hr).

Across the hospitalizations, pleural fluid bacterial cultures and acid-fast bacilli smears were negative, procalcitonin remained negative, and blood cultures showed no evidence of infection. Repeated pleural fluid cytology demonstrated reactive mesothelial cells, histiocytes, and inflammatory cells without malignant cells. CT imaging of the chest, abdomen, and pelvis demonstrated no evidence of an underlying malignancy.

The pigtail catheter was removed after four days without immediate reaccumulation of the pleural effusion. Serial chest radiographs again demonstrated marked improvement of the left pleural effusion following pigtail catheter drainage (Figure 2E, 2F). Dyspnea and hypoxemia resolved, and the patient remained stable on room air. CRP declined further to 22.5 mg/L (<8 mg/L) on day 12 of admission. The patient was discharged home on room air with plans to continue colchicine for three months. Tables 2, 3 summarize the clinical timeline and pericardial and pleural fluid characteristics.

**Table 2:** Clinical timeline. EF, ejection fraction; RA, right atrial; RV, right ventricular; TTE, transthoracic echocardiography

Hospital day	Clinical event	Key findings/intervention
Initial procedure	Amulet implantation	28-mm device; appropriate position; no peridevice leak; no immediate pericardial effusion
Hospitalization no. 1 - day 1	Chest pain, dyspnea, hypoxemia	Multilead ST elevations; negative troponins; large pericardial effusion with tamponade; CRP 78.8 mg/L
Hospitalization no. 1 - day 1	Pericardiocentesis	780 mL frank blood removed
Hospitalization no. 1 - day 3	Early post-drainage evaluation	CRP peaked at 103.5 mg/L; CT showed large left pleural effusion and small residual pericardial effusion
Hospitalization no. 1 - days 6 and 9	Pleural drainage	Recurrent large left exudative pleural effusion; high-dose aspirin/colchicine treatment; CRP declined to 13.9 mg/L
Hospitalization no. 1 - day 18	Discharge	Discharged to skilled nursing facility on colchicine
Hospitalization no. 2 - day 5	Recurrent pleural effusion	CRP 105.9 mg/L; EF 61.6%; normal RV function and estimated RA pressure 3 mmHg
Hospitalization no. 2 - day 6	Thoracentesis	1,500 mL serosanguineous exudative fluid removed
Hospitalization no. 2 - day 9	Discharge	Colchicine not continued at discharge
Hospitalization no. 3 - day 1	Third presentation	Dyspnea/tachycardia; large left pleural and moderate pericardial effusions; CRP 87.8 mg/L
Hospitalization no. 3 - day 2	Colchicine restarted	Planned three-month course; TTE showed small circumferential pericardial effusion without tamponade
Hospitalization no. 3 - day 6	Pigtail drainage	Lymphocyte-predominant exudative pleural fluid; CRP 56.1 mg/L
Hospitalization no. 3 - day 12	Discharge	CRP 22.5 mg/L; asymptomatic and discharged home on room air

**Table 3:** Serial pericardial and pleural fluid characteristics. Reference ranges not established for body fluid cell counts, differential, or chemistries. Serum protein (6-8 g/dL) and LDH (150-220 U/L) provided in the table if available. E, eosinophils; L, lymphocytes; LDH, lactate dehydrogenase; M, monocytes; N, neutrophils

Hospital day	Fluid	Appearance	RBC/µL	Nucleated cells/µL	Protein (g/dL)	LDH (U/L)	Differential
Hospitalization no. 1 - day 1	Pericardial	Red/bloody	700,000	1,389	5.5 (6.8 g/dL)	2,344	N 33%, L 34%, M 32%, E 1%
Hospitalization no. 1 - day 6	Pleural	Yellow/hazy	1,243	2,010	2.9 (6.5 g/dL)	746	N 63%, L 15%, M 11%, mesothelial 12%
Hospitalization no. 1 - day 9	Pleural	Orange/turbid	51,645	1,100	3.1	-	N 3%, L 47%, M 8%, mesothelial 41%, E 1%
Hospitalization no. 2 - day 6	Pleural	Red/cloudy	93,748	378	3.1 (6.4 g/dL)	141 (193 U/L)	N 6%, L 45%, M 21%, mesothelial 17%, E 11%
Hospitalization no. 3 - day 6	Pleural	Yellow/colorless, turbid	16,879	151	3.1 (6.2 g/dL)	178 (147 U/L)	N 11%, L 79%, M 2%, mesothelial 8%

## Discussion

This case presents recurrent pleuropericardial inflammation following Amplatzer Amulet LAAO. Several characteristics strongly suggest PCIS as the underlying diagnosis: a clear temporal association with cardiac instrumentation; chest pain accompanied by multilead ST-segment elevations and negative troponin levels; hemorrhagic pericardial tamponade; subsequent recurrent exudative pleural effusions; recurrence of pericardial effusion; repeatedly elevated inflammatory markers; and an inconclusive evaluation for infection and malignancy.

PCIS represents an inflammatory response occurring after injury to the pericardium, myocardium, or adjacent structures. Proposed diagnostic criteria require at least two of five findings: fever without an alternative cause, pleuritic or pericarditic chest pain, pericardial or pleural friction rub, pericardial effusion, and pleural effusion accompanied by elevated CRP [2,3]. Our patient met at least three criteria: pericarditic chest pain, pericardial effusion, and pleural effusion with markedly elevated CRP. He did have one mildly elevated temperature of 99.1°F on day 6 of his third hospitalization, but no other fevers were documented during any of his hospitalizations. The accompanying electrocardiographic changes and response of inflammatory markers to treatment provided additional evidence of pericardial inflammation.

The timing and nature of the initial effusions deserve particular consideration. A TEE immediately after Amulet implantation demonstrated appropriate device positioning, no peridevice leak, and no pericardial effusion. Nevertheless, approximately 11 days later, the patient had a large hemorrhagic effusion with tamponade. Delayed pericardial effusions following LAAO are increasingly recognized. In a prospective series of 1,023 attempted LAAO procedures, 44 patients (4.3%) developed pericardial effusion; 34 occurred within seven days and 10 between eight and 365 days [2]. More recently, analysis of the US Food and Drug Administration Manufacturer and User Facility Device Experience database identified 290 reported pericardial effusions associated with Amulet devices. Among those reported Amulet-associated effusions, 99 (34.1%) occurred after hospital discharge, and more than half of those late events required an intervention [10]. Importantly, the passive surveillance database lacks a denominator of implanted devices; therefore, these percentages describe reported pericardial effusion cases and cannot be interpreted as population incidence or used to directly compare device-specific risk.

A plausible mechanism in this case is minor procedural injury or device-related microtrauma resulting in hemopericardium, which subsequently triggered an inflammatory pleuropericardial response. Blood within the pericardial space is recognized as a potential inflammatory stimulus in PCIS [3]. A remarkably similar syndrome has been reported following Watchman LAAO: a patient developed recurrent unilateral left exudative pleural effusion, chest discomfort, pericardial effusion, and elevated CRP after device implantation. The authors proposed fixation-anchor microperforation followed by an inflammatory response analogous to post-pericardiotomy syndrome [6]. Watchman and Amulet are the two most frequently used percutaneous LAAO devices globally and differ in device design and mechanism of appendage sealing. In a 2023 meta-analysis of three randomized trials including 2,150 patients, overall mortality, stroke, systemic embolism, and major bleeding were comparable, although Amulet was associated with more procedure-related complications and fewer peridevice leaks than Watchman [1]. These comparative data do not establish whether the risk of PCIS differs between devices.

Our case differs in the dramatic initial presentation with frank hemorrhagic tamponade and in the repeated recurrence of the pleural effusion over several months. Recurrent pericarditis is well recognized after an initial episode of acute pericarditis, but recurrent pleuropericardial disease specifically following LAAO is sparsely described. Contemporary pericarditis guidance reports recurrence after approximately 15-30% of initial pericarditis episodes, with higher recurrence rates after a first recurrence; however, these data are not specific to PCIS or LAAO [5,11]. The limited published experience with recurrent pleuropericardial inflammation after LAAO therefore makes the repeated pleural and pericardial recurrences in our patient particularly noteworthy. Figure 3 contains additional images from the patient’s clinical course.

**Figure 3:**
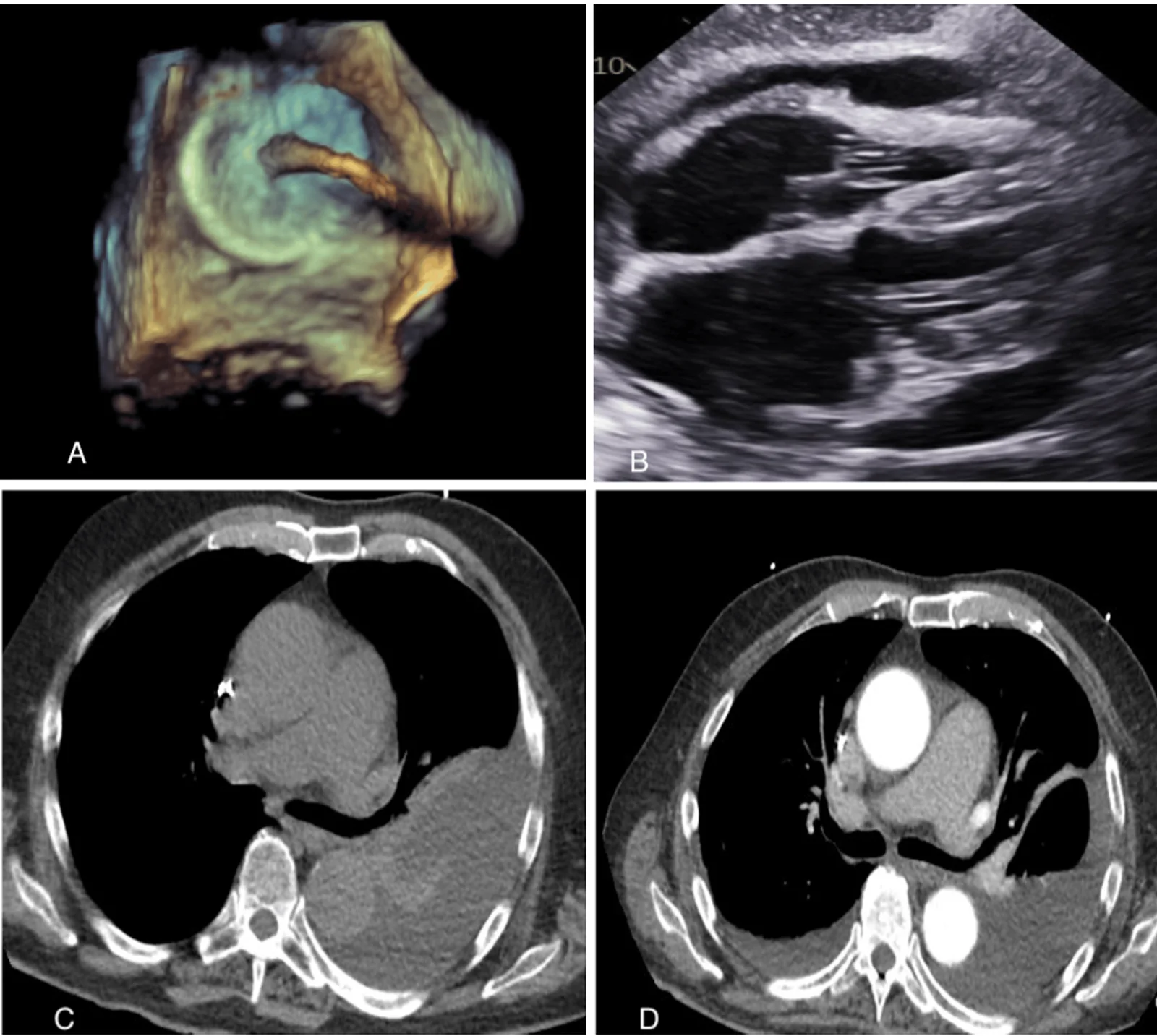
Evolution of pleuropericardial disease following Amplatzer Amulet implantation. (A) Post-implant 3D TEE image demonstrating the appropriately positioned Amulet device. No pericardial effusion was present on post-implant imaging. (B) 2D TTE image demonstrating the large circumferential pericardial effusion during the first hospitalization. (C) CT image demonstrating the large left pleural effusion during the first hospitalization. (D) Contrast-enhanced CT image demonstrating recurrent large left pleural effusion and recurrent pericardial effusion during the third hospitalization. TEE, transesophageal echocardiography; TTE, transthoracic echocardiography

Direct mechanical complications of the Amulet device remain an important alternative or potentially complementary mechanism. Delayed erosion of an Amulet device into the pulmonary artery has been reported six months after implantation, resulting in hemorrhagic tamponade [7]. Another case documented LAA erosion at two locations corresponding to the Amulet disc and lobe 14 months after implantation [8]. Delayed hemorrhagic pericardial effusion has also been described months after Amulet implantation, with device-associated microperforation and subsequent inflammation proposed as a potential mechanism [9]. Thus, mechanical injury and PCIS should not necessarily be viewed as mutually exclusive: a small device-related injury could provide the initial stimulus for a subsequent inflammatory syndrome.

A limitation of the present case is that a dedicated cardiac CT or repeat TEE was not performed following the development of the effusions. Consequently, device-related microperforation or erosion could not be definitively excluded. The absence of pericardial effusion on immediate post-implantation TEE argues against a readily apparent acute perforation but does not exclude subsequent microperforation or erosion. Meeting CLOSE criteria confirms satisfactory device compression, lobe orientation, separation from adjacent structures, and sealing at the time of implantation but does not exclude microscopic tissue injury or delayed device-tissue interaction. Accordingly, the absence of an immediate pericardial effusion and satisfactory device positioning do not preclude small-volume procedural or device-related injury to the thin left atrial appendage wall. Such injury could produce hemopericardium and serve as the inflammatory trigger for subsequent PCIS without causing an immediately apparent macroperforation.

Several alternative explanations for the recurrent pleural effusion were considered. The patient had heart failure with preserved ejection fraction; however, the persistent unilateral nature of the effusion, its repeated classification as exudative, and the August echocardiographic findings argued against decompensated heart failure as the primary explanation. At that time, left ventricular ejection fraction was 61.6%, right ventricular size and systolic function were normal, and estimated right atrial pressure was only 3 mmHg (0-5 mmHg) despite the presence of a sizable left pleural effusion.

Infection was also unlikely. Blood cultures, pleural microbiologic studies, acid-fast bacilli smears, and serial procalcitonin measurements were negative. Similarly, repeated cytologic examinations demonstrated no malignant cells, CT imaging of the chest, abdomen, and pelvis revealed no evidence of malignancy, routine cancer screening was current, and cystourethroscopy was normal despite a remote reported history of bladder cancer. The evolution of the pleural fluid from an initially neutrophil-predominant effusion to a progressively lymphocyte-predominant effusion, culminating in 79% lymphocytes during the final hospitalization, was also compatible with an evolving inflammatory process, although this finding is not specific for PCIS.

The clinical course surrounding colchicine provides additional, although not definitive, support for an inflammatory mechanism. During the initial hospitalization, CRP declined from a peak of 103.5 mg/L (<8 mg/L) to 13.9 mg/L during treatment with high-dose aspirin and colchicine 0.6 mg twice a day. The medication course also highlights the importance of clear communication regarding the intended duration of anti-inflammatory therapy and careful medication reconciliation during transitions between acute hospitalization, skilled nursing care, and home. Following the initial hospitalization, no predetermined stop date for colchicine was documented, as treatment duration was intended to be reassessed at outpatient cardiology follow-up. The patient remained in skilled nursing care and was rehospitalized before that follow-up occurred, complicating continuity of the anti-inflammatory regimen. Colchicine was not included in the discharge medication regimen following the second hospitalization. In this case, the medical record did not establish when or why colchicine was discontinued after the initial hospitalization. Approximately 10 days later, the patient returned with both a large recurrent left pleural effusion and recurrent pericardial effusion, accompanied by a CRP of 87.8 mg/L (<8 mg/L). After colchicine was restarted and the pleural effusion was drained, CRP declined to 56.1 mg/L and subsequently to 22.5 mg/L by discharge. Symptoms and hypoxemia resolved. Because pleural drainage occurred concurrently with anti-inflammatory treatment, the improvement cannot be attributed to colchicine alone. Nevertheless, the pattern of recurrence and improvement further supports an inflammatory component.

Treatment duration specifically for PCIS following LAAO has not been established. Contemporary pericarditis guidelines recommend colchicine in addition to aspirin or an NSAID for approximately three months following a first episode of acute pericarditis and for at least six months in recurrent or incessant pericarditis [11]. The 2025 American College of Cardiology guidance similarly recommends three months for acute pericarditis and six to 12 months for recurrent disease [5]. Our patient was ultimately prescribed a three-month course following the final hospitalization, with plans for cardiology follow-up prior to the end of treatment for reevaluation.

Management options for refractory recurrent pericarditis have also expanded with therapies targeting IL-1. Contemporary guidance supports IL-1 blockade, including anakinra or rilonacept, in selected patients with recurrent or incessant pericarditis who fail first-line anti-inflammatory therapy, particularly when an inflammatory phenotype with elevated CRP persists [5,11,12]. These therapies were not required in our patient but may represent an option for refractory or multiply recurrent PCIS. Whether patients with recurrent PCIS following LAAO respond similarly to populations studied in recurrent idiopathic pericarditis remains uncertain and warrants further study.

This case has several clinical implications. First, recurrent dyspnea after LAAO should prompt evaluation not only for device-related pericardial complications but also for pleural disease. Second, PCIS can manifest with prominent pleural involvement and may recur after the initial pericardial effusion has resolved. Third, an inflammatory syndrome does not eliminate the possibility of underlying device-related microtrauma; mechanical erosion or perforation should remain in the differential, particularly when the initial pericardial fluid is hemorrhagic. Finally, longitudinal assessment of symptoms, inflammatory markers, and both pleural and pericardial imaging may help identify this uncommon postprocedural syndrome. Although demographic and procedural risk factors for various PCISs have been described [2], the role of age in PCIS following percutaneous LAAO is not well established.

## Conclusions

PCIS should be considered in patients who develop recurrent pleural and pericardial effusions following LAAO, particularly when accompanied by pleuritic or pericarditic symptoms and elevated inflammatory markers. In this case, hemorrhagic tamponade shortly after Amplatzer Amulet implantation was followed by recurrent unilateral exudative pleural effusions, recurrent pericardial effusion, and marked systemic inflammation despite an unrevealing infectious and malignant evaluation. The clinical course was most consistent with PCIS, potentially triggered by procedural or device-related pericardial injury.

Because delayed device erosion or microperforation may present similarly and may itself initiate an inflammatory response, PCIS and mechanical injury should not be viewed as mutually exclusive. Recognition of this relationship is important because patients presenting with recurrent effusions after LAAO require evaluation for both potentially serious mechanical complications and treatable post-cardiac inflammatory disease. Further study is needed to define optimal diagnostic criteria, treatment duration, and the role of targeted anti-inflammatory therapies in recurrent PCIS following LAAO.
